# Facial shape affects self-perceived facial attractiveness

**DOI:** 10.1371/journal.pone.0245557

**Published:** 2021-02-03

**Authors:** Georgios Kanavakis, Demetrios Halazonetis, Christos Katsaros, Nikolaos Gkantidis

**Affiliations:** 1 Department of Orthodontics and Dentofacial Orthopedics, Tufts University School of Dental Medicine, Boston, Massachusetts, United States of America; 2 Department of Pediatric Oral Health and Orthodontics, University Center for Dental Medicine, University of Basel, Basel, Switzerland; 3 Department of Orthodontics, School of Dentistry, National and Kapodistrian University of Athens, Athens, Greece; 4 Department of Orthodontics and Dentofacial Orthopedics, University of Bern, Bern, Switzerland; University of Zurich, SWITZERLAND

## Abstract

Facial appearance expresses numerous cues about physical qualities as well as psychosocial and personality traits. Attractive faces are recognized clearly when seen and are often viewed advantageously in professional, social and romantic relationships. On the other hand, self-perceived attractiveness is not well understood and has been mainly attributed to psychological and cognitive factors. Here we use 3-dimensional facial surface data of a large young adult population (n = 601) to thoroughly assess the effect of facial shape on self-perceived facial attractiveness. Our results show that facial shape had a measurable effect on self-perception of facial attractiveness in both sexes. In females, self-perceived facial attractiveness was linked to decreased facial width, fuller anterior part of the lower facial third and more pronounced middle forehead and root of the nose. Males favored a well-defined chin, flatter cheeks and zygomas, and more pronounced eyebrow ridges, nose and middle forehead. The findings of this study support the notion that self-perceived facial attractiveness is not only motivated by psychological traits, but objectively measured phenotypic traits also contribute significantly. The role of social stereotypes for facial attractiveness in modern society is also inferred and discussed.

## Introduction

Visual contact with a human face provides basic identifying information regarding age, gender and ethnic background, but also triggers a series of opinion-forming decisions about a person’s attractiveness, physical health, personality and more [1–5]. Such decisions tend to be formed in less than a second and change very little as the observation time increases [6] thus, confirming the common belief that a first impression is very important in human interactions. Although purely a physical trait, facial appearance plays a broader role in shaping public opinion about an individual’s trustworthiness, mating quality, intelligence and academic competence [7–10], and thus, it may shape various life experiences, such as personal and romantic relationships and professional success [11, 12]. The structure of the face, the smile, the eyes and the shape of the lips are features that contribute to facial attractiveness as perceived by others [11]. Due to the effect of physical features on facial attractiveness, the notion that “beauty is in the eye of the beholder” is often scrutinized, and a more global perception of attractiveness has been discussed [9].

Despite the extensive research in this field, there is little focus on self-perceived facial attractiveness. We know that it is strongly linked to self-confidence and self-esteem [13, 14], and that it may influence individual sexual preferences in females [15]. However, there is no information regarding physical features that may influence personal appreciation of facial attractiveness. Here, we explore and discuss the effect of facial shape on self-perceived facial attractiveness, in a large socially homogenous, but ethnically diverse sample of young adults. Different sexes were tested separately. As a secondary hypothesis, we also addressed the main question within ethnic subgroups, to test the robustness of the findings within these groups. The results of this study add to the existing body of literature exploring the factors that contribute to human perception of facial appearance.

## Material and methods

### Ethical approval

This cross sectional, observational investigation was reviewed and approved by the Health Sciences Institutional Review Board (IRB) of Tufts University in Boston, Massachusetts (IRB#: 11181). Written consent was received from all participants prior to participation in this investigation.

### Population

The study population consisted of 613 (214 males; 399 females) volunteers, all pre-doctoral medical or dental students at Tufts University in Boston, Massachusetts, who responded to an on-campus advertisement. All participants were young adults, aged 21 to 35 years, who were raised in the United States and spoke English as their native language. Each participant was compensated with a gift card for $20 for partaking in the study. Individuals who presented craniofacial syndromes, visible deformations of the face or had a history of facial plastic surgery, were excluded. After recruitment and participation, data were also excluded on the basis of sexual orientation, due to reported differences in appreciation of facial attractiveness [16]. As a result, our final study sample consisted of 601 heterosexual young adults (208 males; 393 females) with various ethnic backgrounds. Demographically, the sample included the following subgroups: 368 Whites (149 males and 219 females), 22 Black (3 males and 19 females), 123 Asians (39 males and 84 females), 71 Indian Asians (14 males and 57 females) and 21 multiracial individuals (4 males and 17 females). Sex, ethnicity and sexuality were self-reported by the participants.

### 3D facial image acquisition

A stereophotogrammetry system (3dMD, Atlanta, USA) was used to capture a three-dimensional facial image of each participant in resting position. Subjects were positioned at a fixed distance of approximately 100 cm from the camera unit, with their head slightly raised (10 degrees) according to the camera guidelines (upright head position). To ensure standardization, participants were seated on a chair placed at the correct distance, with their upper body in a comfortable upright position and their back resting on the back of the chair. They were asked to maintain the upright head position, which was also adjusted by an experienced research team member, if needed. In order to acquire the image at rest, subjects were instructed to keep their teeth in slight contact, their lips in a resting position, without straining, and their eyes open without stretching the forehead.

### Facial shape definition

**Fig 1:**
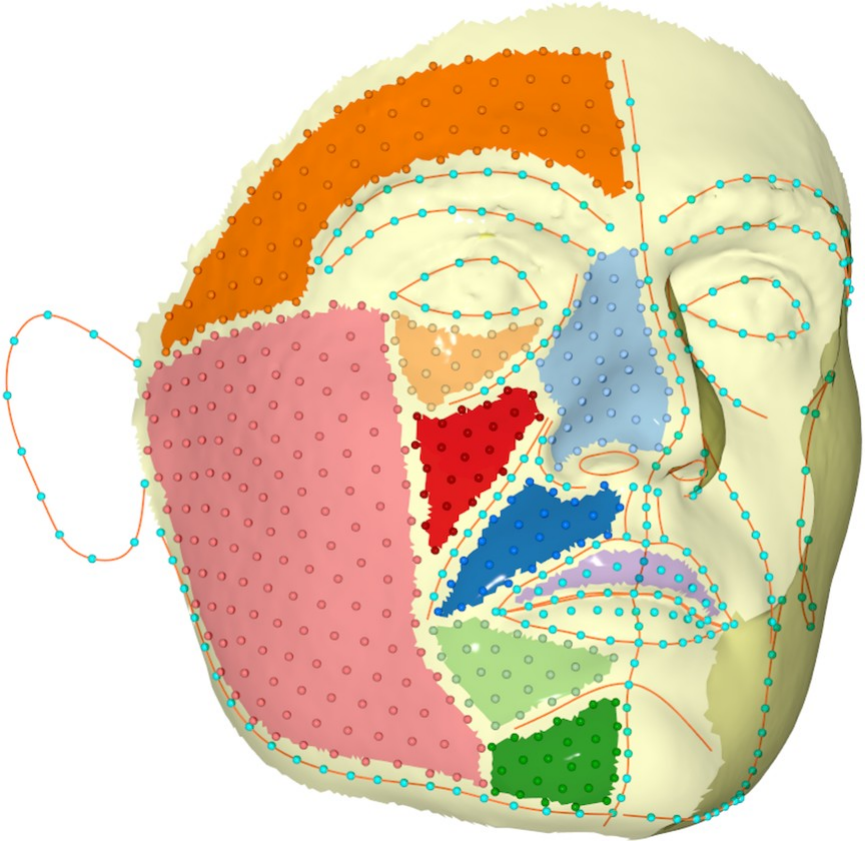
Distribution of all 1021 landmarks and semi-landmarks on the facial surface. Semi-landmarks were initially positioned to depict various areas of the face but were allowed to slide along curves (curve semi-landmarks) or the surface (surface semi-landmarks) during TPS (Thin Plate Spline) transformation. In order to avoid clutter, surface semi-landmarks are only depicted on one side of the face.

Three-dimensional surface images were imported in “ViewBox 4.1” software (dHAL Software, Kifissia, Greece) for digitization and application of geometric morphometric methods. A similarly acquired facial image of a person who did not participate in the study, was used as reference shape (template) to place landmarks and semi-landmarks [17]. In total, the facial surface was described by 1021 landmarks and semi-landmarks (Fig 1).

The digitization was performed semi-automatically, first by manually drawing curves on anatomical structures of the face and, subsequently, by automatically adding semi-landmarks at equidistant positions on the curves and at uniform locations on the surface of the face, delimited according to the manually drawn curves and specific fixed landmarks. The latter corresponded to fixed points of the local anatomy, such as the anatomical limits of eyes (inner and outer canthion) and the mouth (inner and outer stomion), were manually placed and considered as traditional landmarks that remained fixed throughout all the stages of the analysis (see below). The other curve landmarks, as well as the surface landmarks, were allowed to slide on their corresponding curves or the facial surface, respectively, and were thus treated as semi-landmarks [18]. Fixed landmarks and curve semi-landmarks were placed first and were used to guide the placement of all the surface semi-landmarks according to the reference shape. After all images were digitized and saved, semi-landmarks were allowed to slide along their related curve or the entire surface, in order to reduce variation in their initial positions produced by the initial equidistant placement of curve- and surface- semi-landmarks, respectively. Sliding was performed to minimize TPS (Thin Plate Spline) bending energy between each separate landmark configuration and the average facial shape of the entire sample [18]. Six cycles of sliding and re-projecting were repeated until no meaningful change in bending energy was observed. This iterative process was performed three times to ensure convergence. The resulting landmark configurations were considered to be homologous representations of the facial surfaces and comprised the final sample for all following shape analyses. A Generalized Procrustes Superimposition was used to superimpose landmark configurations and transform landmark coordinates to Procrustes coordinates [19, 20]. In order to reduce the number of shape variables (Procrustes coordinates) for subsequent statistical analyses, a Principal Component Analysis (PCA) was performed and shape variation was explored with shape space principal components (PCs). Facial shapes were morphed using TPS transformations [21], in order to visualize extremes of relevant PCs. In addition, extreme values of self-perceived facial attractiveness (dependent variable) were also visualized through morphing of average facial shapes.

### Assessment of facial attractiveness

Following 3D facial image acquisition, each individual was asked to complete a questionnaire without looking at any personal pictures or images. The questionnaire consisted of 6 units and took approximately 15 minutes to complete. For the needs of the present study, we used the following question belonging to the facial attractiveness unit that was the first to be answered after the demographic information: “*How would you rate the esthetic appearance of your face*?*”* (S1 Fig). The answer was recorded in a 100 mm VAS (visual analogue scale) [22]. Participants completed the questionnaires privately, without any immediate supervision and were unaware of the study aim. A research team member was in discrete proximity in order to answer any questions that participants might have during the process. Participants’ scores were recorded in an Excel sheet (Microsoft Excel, Microsoft ^**©**^, Richmond WA, USA) by an operator who was not involved in any other part of the study and was blinded regarding the purpose of the study. To record VAS scores, this operator converted participants’ markings into Euclidean metric variables by measuring the distance of the mark on the VAS scale from the start of the 100 mm line with a digital caliper.

### Error of the method

In order to evaluate the reliability of the digitization process, forty randomly selected images were digitized twice, by two calibrated operators. The two digitizations were done at least 2 weeks apart. Interrater reliability was evaluated by measuring the mean Procrustes distance between the two independent digitizations [23]. Intra-rater reliability was tested by calculating the percentage of variance in PC scores explained by the difference between the two digitizations.

To assess the reliability of the self-evaluation scores, 93 participants agreed to return for a second visit, at least 1 month after the first, during which the same original research process was repeated. Bland-Altman plots were used to test the repeatability of their answers.

Bland Altman plots were also used to explore the error during data entry; the operator who inserted the questionnaire scores into an Excel sheet, as described above, repeated the entire process at a second time point at least 1 month later for 50 randomly selected subjects.

### Statistical analyses

Sexual shape dimorphism was assessed by calculating the Procrustes distance between group means, using permutation tests to determine its significance. The relationship between self-perceived facial attractiveness and facial shape was tested with multivariate linear regression after all necessary assumptions were tested and met (see S2 Fig). Facial shape was described through 9 face shape PCs (stemming from the Procrustes coordinates of all landmarks) that explained more than 70% of the variation within the entire sample (S3 Fig). Comparisons of self-perceived facial attractiveness scores within the entire sample and within subgroups were performed with independent t-tests. All statistical analyses were performed with “ViewBox 4.1” software and IBM SPSS statistics for Windows (Version 26.0. Armonk, NY: IBM Corp.) The level of statistical significance was set at p = 0.05.

## Results

### Final sample and outliers

Shape outliers and extreme VAS values were reviewed prior to inclusion in the final sample, in order to exclude the possibility of manual errors in the digitization process and in transferring participant answers from the questionnaires to an excel sheet. No errors were detected and thus it was decided to include outliers in all further analyses as they represented true data.

### Error calculation

The mean Procrustes distance between digitizations performed by two independent operators was assessed through permutation tests (10,000 permutations) and was found to be minimal, indicating a high inter-rater reliability (Procrustes distance = 0.019; P = 0.196).

In addition, the 9 first PCs (explaining 70.2% of total variation) of two consecutive digitizations were used to calculate random digitization error, as percentage of total shape variance in shape space. This resulted in a random error of 6.9%, indicating good intra-rater reliability.

The repeatability of subjective evaluations of self-perceived attractiveness was assessed with a Bland-Altman plot and was considered acceptable for the specific outcome (Mean difference: -0.2; Lower 95% limit of agreement: -18.6; Upper 95% limit of agreement: 18.2) (S4 Fig). The error stemming from recording participants’ answers was negligible (Mean difference: 0.3; 95% LoA: -0.6 to 1.2).

### Shape analyses

**Fig 2:**
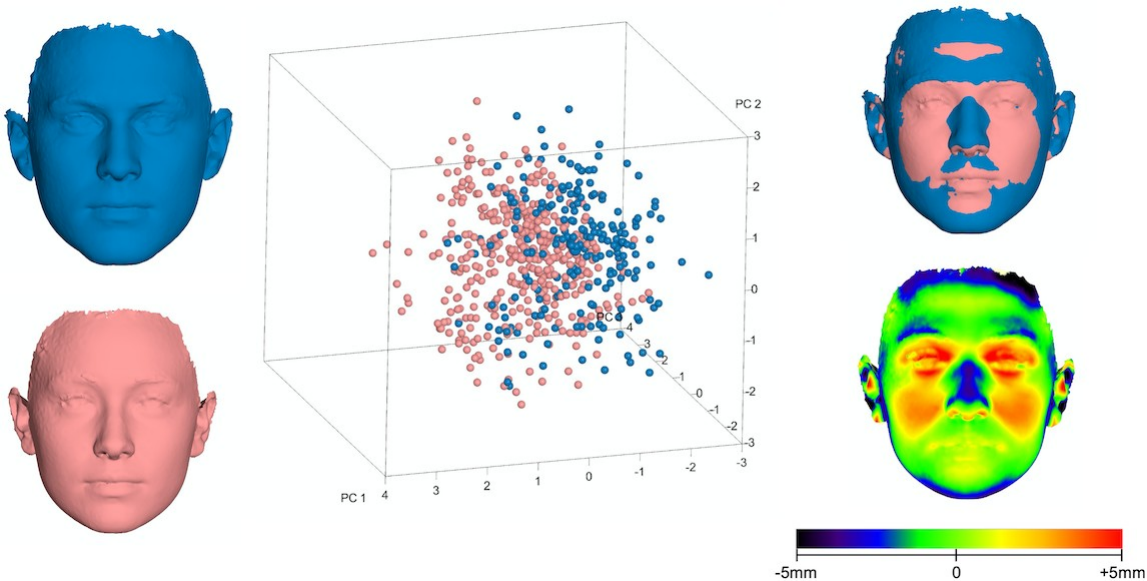
3D PCA graph displaying facial variation according to sex (in SD units), in the entire sample, as explained by PC1 (20.9%), PC2 (15.1%) and PC3 (9%). The corresponding 3D facial morphings represent the average female (red) and male (blue) facial shapes. A best-fit superimposition (upper right) reveals the surface differences in facial shape between males and females and the color-map (lower right) displays the magnitude of those differences, as distance of the female from the male face (positive: forward).

Facial shape differences were assessed with Procrustes Superimposition and subsequent comparisons between shape coordinates; and were visualized with surface superimpositions and color maps. There was significant sexual shape dimorphism between female (n = 393) and male groups (n = 208), assessed through permutation tests on the mean Procrustes distance (Procrustes distance: 0.028, P<0.001; 10,000 permutations). Shape variation between males and females is presented in a 3D plot as well as using surface transformations of the average male and average female face (Fig 2). TPS surface transformations were created by warping the surface of one male and one female individual (that were both relatively close to the sample average) according to the average landmark configurations of the male and female groups, respectively. Surface superimposition (iterative closest point-ICP best fit approach under the following settings: 100% estimated overlap of meshes, matching point to plane, exact nearest neighbor search, 100% point sampling) [24] of the average male and average female face revealed that males presented wider faces, more prominent eyebrow ridges, a more defined mandible with larger chin projection, flatter lips and a more pronounced nose (Fig 2). Due to the broad inherent sexual differences including sexual dimorphism in facial shape, the latter variable and its effect on self-perceived facial attractiveness was studied separately in males and females.

**Fig 3:**
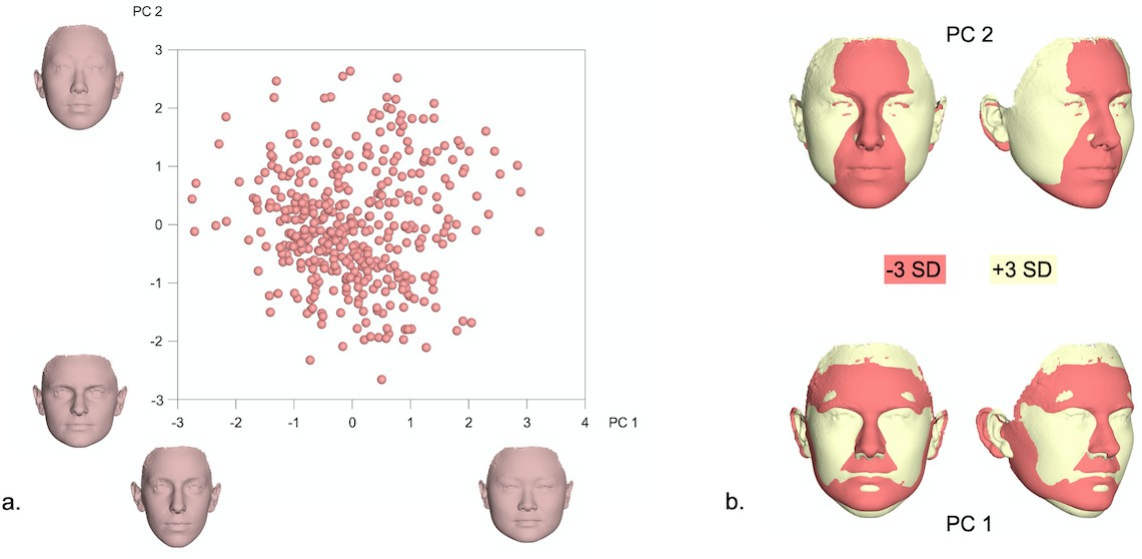
a. PCA graph displaying facial shape variation in females (in SD units), as explained by PC1 (21%) and PC2 (15.1%). The corresponding 3D facial morphings show the shape extremes from -3 to +3 standard deviations of PC scores within each axis. b. Best fit superimpositions of shape transformations created from the Procrustes coordinates corresponding to -3SD and +3SD of PC scores within each axis from the average shape configuration in the female population. The superimpositions display the direction of shape variation explained by each PC.

**Fig 4:**
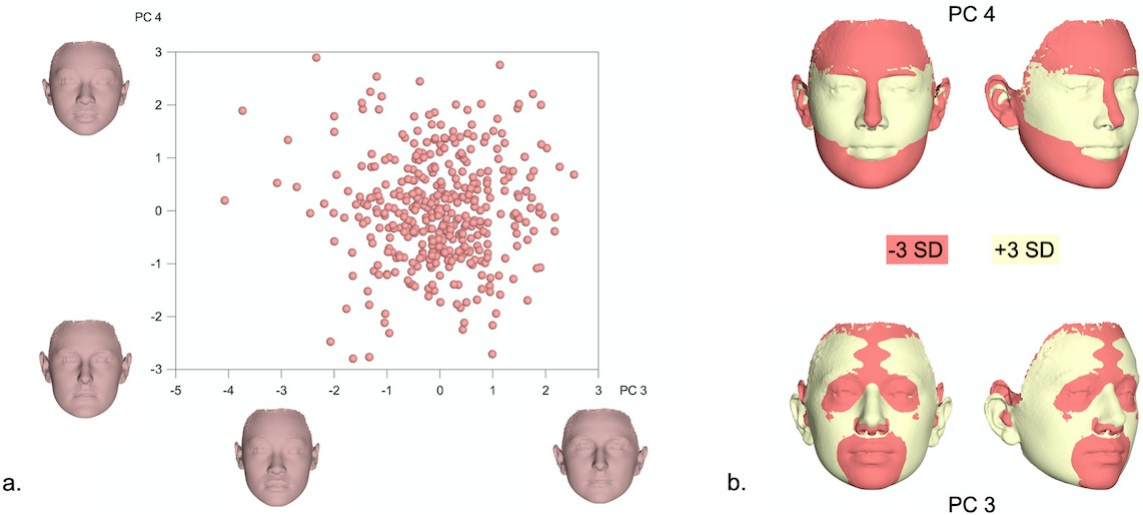
a. PCA graph displaying facial shape variation in females (in SD units), as explained by PC3 (9.1%) and PC4 (7.2%). The corresponding 3D facial morphings show the shape extremes from -3 to +3 standard deviations of PC scores within each axis. b. Best fit superimpositions of shape transformations created from the Procrustes coordinates corresponding to -3SD and +3SD of PC scores within each axis from the average shape configuration in the female population. The superimpositions display the direction of shape variation explained by each PC.

Principal Components PC1-PC4, explaining more than 50% of total variation (PC1: 21%, PC2: 15.1%, PC3: 9.1%, PC4: 7.2%) were used to visualize female facial shape variation (Figs 3 and 4). As depicted in Fig 3, for example, PC1 primarily described the vertical dimension and the facial convexity, while PC2 was mainly related to the width and the anteroposterior length of the face. Similarly, PC3 mostly regarded the flatness of the lower third of the face, including the shape of the lips and PC4 was linked to facial roundness and convexity.

**Fig 5:**
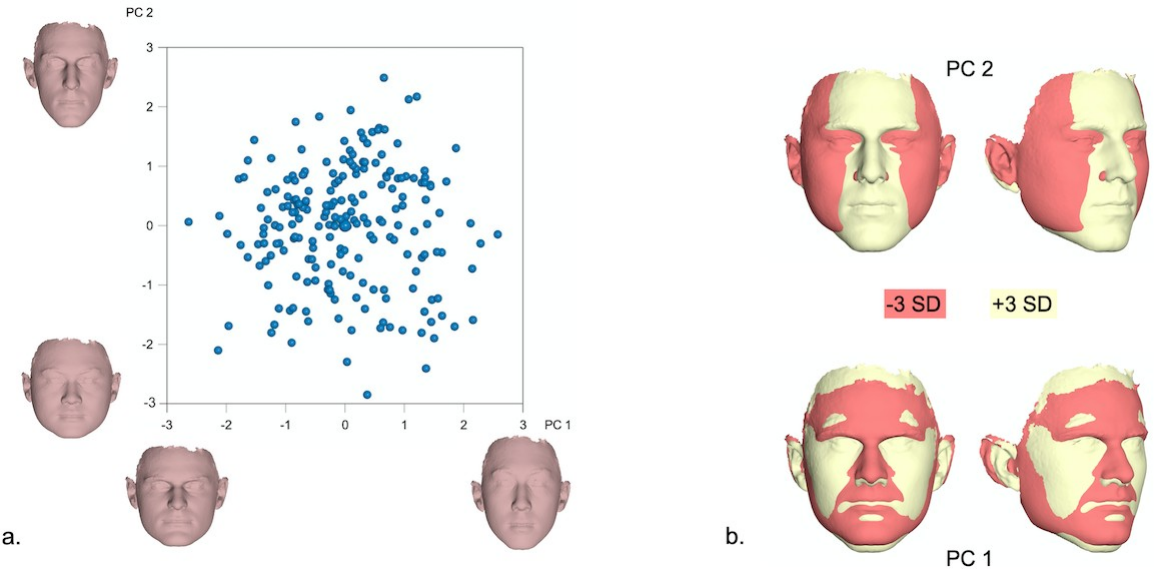
a. PCA graph displaying facial shape variation in males (in SD units), as explained by PC1 (20%) and PC2 (16.8%). The corresponding 3D facial morphings show the shape extremes from -3 to +3 standard deviations of PC scores within each axis. b. Best fit superimpositions of shape transformations created from the Procrustes coordinates corresponding to -3SD and +3SD of PC scores within each axis from the average shape configuration in the male population. The superimpositions display the direction of shape variation explained by each PC.

**Fig 6:**
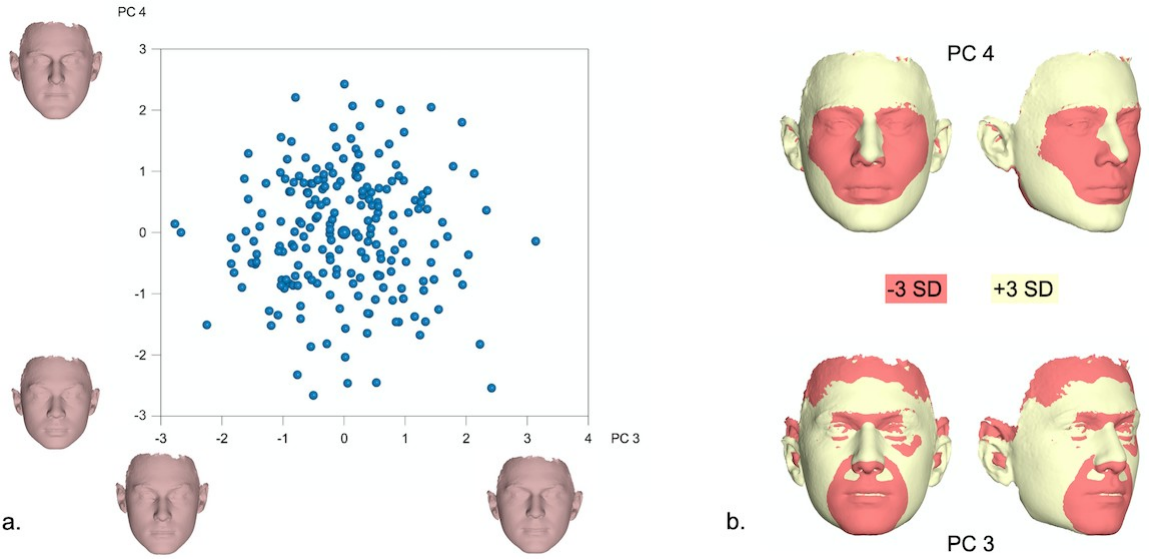
a. PCA graph displaying facial shape variation in males (in SD units), as explained by PC3 (8.1%) and PC4 (6.3%). The corresponding 3D facial morphings show the shape extremes from -3 to +3 standard deviations of PC scores within each axis. b. Best fit superimpositions of shape transformations created from the Procrustes coordinates corresponding to -3SD and +3SD of PC scores within each axis from the average shape configuration in the male population. The superimpositions display the direction of shape variation explained by each PC.

Facial shape variation in males is exhibited in Figs 5 and 6, again, through PC1-PC4, also explaining more than 50% of variation in the male sample (PC1: 20%, PC2: 16.8%, PC3: 8.1%, PC4: 6.3%). As visualized in the warped surface images, PC1 mainly described facial height and convexity, PC2 explained differences in facial width and sagittal length, and both first PCs were related to variation in lip and eye shape. Moreover, PC3 was mostly associated with the flatness of the lower third of the face and PC4 primarily explained differences in facial roundness and convexity.

Facial shape variation within ethnic subgroups of the entire sample is presented as S5–S12 Figs.

### Self-perceived facial attractiveness

There were significant differences in self-perceived facial appearance scores between males and females in the entire group (P<0.001) and among whites, which comprised the most represented subgroup (P<0.001). However, the difference between white and non-white females, which was the second largest subgroup studied, largely approximated the level of significance (P = 0.043). Descriptive and comparative data regarding our primary outcome are displayed in Table 1.

**Table 1:** Comparisons (independent t-tests) of self-perceived facial attractiveness scores within the entire sample and within subgroups.

		N	Self-perceived attractiveness (VAS score)		P-values
			Mean	SD	
Entire sample	Females	393	63.60	12.90	<0.001
	Males	208	67.57	13.20	
Whites	Females	219	64.80	11.81	<0.001
	Males	149	69.35	11.83	
Females	Non-white	174	62.08	14.05	0.043
	White	219	64.80	11.81	

### Facial shape and self-perceived facial attractiveness

#### I. All participants (n = 601)

**Fig 7:**
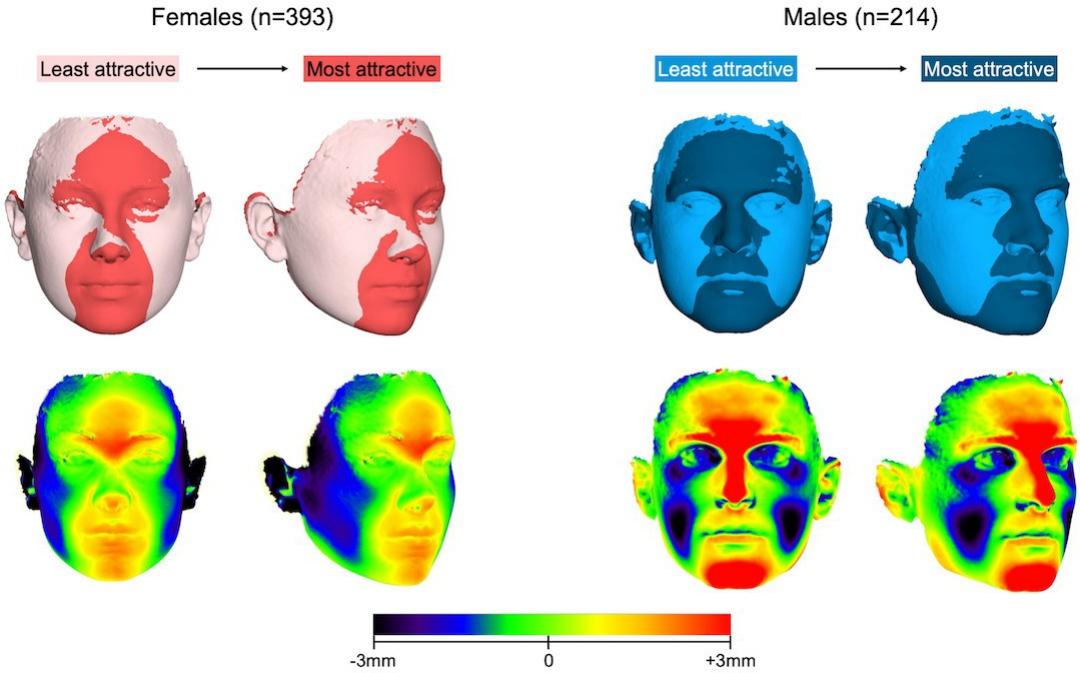
Best fit superimpositions showing the surface differences between the least and most attractive variation of a female (upper row, left) and male (upper row, right) face. The magnitude of difference between the two surface images can be visualized on the color maps (lower row), where distances between the most attractive and the least attractive face are shown (positive: forward).

The effect of facial shape (as defined by the first 9 PCs; 70.2% explained variance) on self-perceived facial attractiveness was significant in both males and females (*P*_males_ = 0.021 and *P*_females_ = 0.004), and predicted 5% and 4% of the variation, respectively (R^2^_males_ = 0.051; R^2^_females_ = 0.039) (S13 Fig). Surface superimpositions of the most and least attractive versions of the female and male face are presented in Fig 7. In females, increased self-perceived facial attractiveness was associated with less facial width and more protruding central part of the face, including fuller lips and a well-defined mid-forehead area. In males, chin protuberance was related to high scores of self-perceived attractiveness, in addition to a relatively square mandible, prominent eyebrow ridges and a pronounced mid-forehead and nasal ridge. Lip fullness, projection of the cheeks and of the area around the eyes had a negative effect on attractiveness scores in males.

#### II. Subgroups analyses

On the basis of the different ethnicities included in the entire male and female groups tested in the study and the size of the individual ethnic groups, it was considered reasonable to assess our primary outcome, also in the subgroup of white male and female individuals, and to further explore the female subgroup by comparing white to non-white females. The results are presented below.

**Fig 8:**
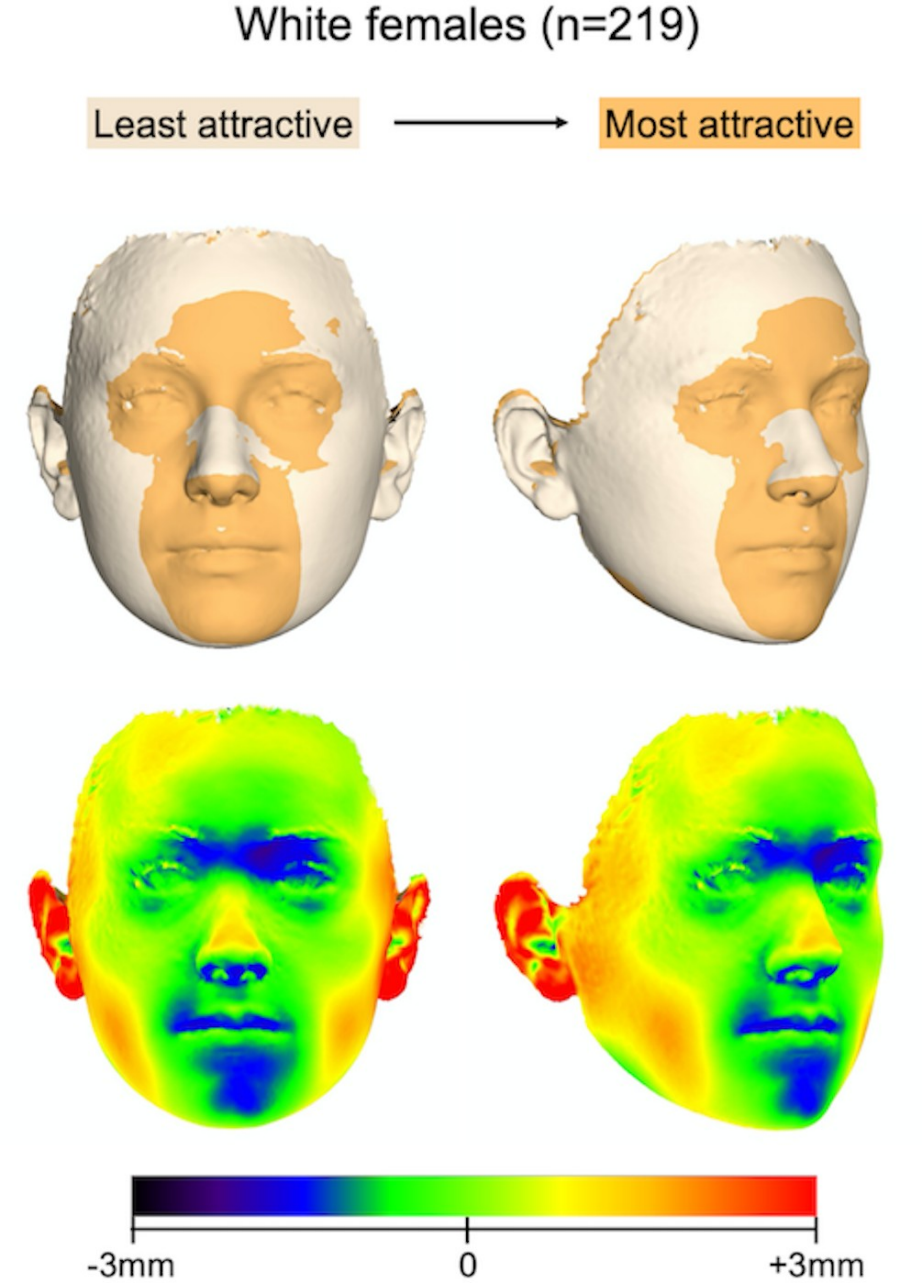
Best fit superimposition showing the surface differences between the least and most attractive variation of a white female (upper row) face. The magnitude of difference between the two surface images can be visualized on the color maps (lower row), where distances between the most attractive and the least attractive face are shown (positive: forward).

*a*. *White participants (n = 368; males*: *149 / females*: *219)*. Facial shape significantly affected self-perceived facial attractiveness in females (*P*_females_ = 0.021) and predicted approximately 5% of the variation in this group (R^2^_females_ = 0.048) (S14 Fig). Fig 8 displays superimpositions of the least and most attractive white female face. It appears that in white women, a narrower face with an increased anteroposterior length and a well-shaped nose ridge area were considered attractive. This pattern is similar to that detected in the total female sample. On the other hand, self-assessments of white males did not seem to be influenced by their facial shape (*P*_males_ = 0.140; R^2^_males_ = 0.032). Thus, the significant effect facial shape had on self-perceived facial attractiveness in the entire male sample, was unnoticeable when white males were tested separately. Also, in white females, facial shape had a slightly stronger effect as compared to the entire female sample. These differences might have cultural and societal implications regarding the perception of attractiveness, which are discussed below.

*b*. *Female participants (n = 393; whites*: *219 / non-whites*: *174)*. The effect of facial shape on self-perceived facial attractiveness in white females is described above. In non-white females, no such effect was evident (*P*_non-white_ = 0.566; R^2^_non-white_ = -0.008). This finding may also be associated to important societal beliefs regarding attractiveness, as discussed further in this manuscript.

## Discussion

This study assesses the effect of facial shape variation on self-perceived facial attractiveness using three-dimensional data of a large young adult population. Current evidence suggests that facial attractiveness, as perceived by others, is related to averageness, symmetry, masculinity/femininity, and also to secondary characteristics, namely skin texture and tone, hair quality and style as well as eye color [9, 11, 12, 25, 26]. Intuitively, it might be expected that objective facial characteristics are only important when judging the esthetic appearance of an unfamiliar face, and not when performing a self-assessment of facial attractiveness. So far, self-perceived facial attractiveness is more commonly linked to internal processes related to individual self-concept and self-esteem [13, 14]. Here, we put this preconception under scrutiny and show that self-perceived facial attractiveness is also affected by objective factors, namely facial shape.

Comparisons of facial shape space (as described by Procrustes coordinates) between males and females in our sample, demonstrated significant sexual dimorphism. Males, on average, presented a wider face with more prominent eyebrow ridges, a pronounced chin and more protruded nose. On the other hand, the average female face was narrower, with more protruded lips and cheeks, as well as more dominant eyes. These facial differences between males and females represent well known phenotypic expressions of sexual hormones during growth and development of the human face [27, 28]. Biologically, they might be related to inherent differences in lung capacity, body mass and distribution of adipose tissue, which to some degree influence facial anatomy [29].

Due to the distinct sexual differences, shape variation was also explored separately in males and females within the present sample. A more careful examination of the principal components explaining more than 50% of the variation within each sex [PC1-PC4] revealed that PC1 and PC2 described changes in the nose, lower facial height and midfacial width (Figs 3–6). Thus, the areas with the largest variation within the male and female population are similar to the areas presenting the largest differences between sexes (Fig 2); potentially signifying the different effect of sexual hormones not only between, but also within same sex populations. A genetic and environmental interpretation of the observed variation might also be plausible. Mapping of the genetic effect on human facial shape has identified a strong genetic control of the lower third of the face (primarily the chin) and the nose [30–32], in all large continental populations. In addition, anatomic investigations of human skulls from populations that lived in diverse climates show marked dissimilarities in the piriform and zygomatic areas between specimens from tropical and temperate areas [33]. These are attributed to evolutionary adaptations to climate conditions and manifest the presence of an additional environmental effect on facial morphology. From a biological view, our results fall within the above spectrum. PC3 and PC4 in our female and male populations, mostly describe changes in the perioral region, the lips and the eyes. The genetic effect on these structures has also been demonstrated, with the distances between the eyes as well as between the eyes and the mouth presenting a high heritability effect [34].

The face is the most influencing factor in human interactions, it contributes to effective communication and affects social and personal relationships [4, 9]. Facial dimorphism related to inherent sexual characteristics plays an important role in romantic relationships by shaping perceived impressions about mating quality, health and reproductive potential [34]. Females, for example, exhibit an increased sexual preference for males with more masculine features during their ovulation period [9, 35]. During this time, females are also judged as more attractive by observers of the same or opposite sex [36].

Features of masculinity and femininity provide cues for physical and social traits, such as attractiveness, personality, trustworthiness, dominance and aggression [5, 8, 9, 12, 34, 37]. Typically, masculine male faces and feminine female faces are considered more attractive by both sexes, although this is only true for small deviations from the average face [11, 12, 37, 38]. Despite the extensive data supporting the above, the notion of universal attractiveness cues has been challenged, and there seem to be significant differences between populations [39]. It is suggested that attractiveness cues are learned within a social environment [37, 39] and, thus, many of our beliefs might be representative of western societies only. In the present study all participants were born and raised in the United States, therefore the ethnic variability within our sample did probably not influence self-assessments considerably.

Our results showed that facial shape had a significant effect on self-perceived facial attractiveness and predicted 4% and 5% of the variation in VAS scores in females and males, respectively. Furthermore, females with more feminine features and males with more masculine features seemed to consider themselves more attractive, confirming the findings of numerous previous studies that have assessed attractiveness with external ratings. Given the multidimensionality of factors interfering with the process of self-assessment, our findings reveal the importance of facial shape, an objective factor, in partially steering peoples’ opinions about themselves. It has been suggested that self-perceived attractiveness is an acquired feature that evolves throughout the course of our lifetime according to our social interactions [40]. Furthermore, it strongly affects romantic relationships; individuals with high self-ratings of attractiveness set higher upper limits in their dating expectations regardless of their objective facial appearance [41]. The effect of facial shape becomes more noteworthy when taking into account that humans evaluate faces that resemble them as 22% more attractive [42]. This fairly narcissistic phenomenon implies that people are less likely to consider their objective appearance when making dating decisions and tend to adjust their attractiveness estimates of their potential dates according to their own appearance. The present study counters this idea, since young adults in our sample appeared to be influenced by the morphology of their faces when making their self-assessment. This is a sign that the intuitive process of making romantic or mating decisions may also be subconsciously influenced by more objective factors, such as an individual’s facial shape.

A more in-depth exploration of our sample, in sub-groups, revealed that in white individuals the previously described effect of facial shape was not evident in males and was stronger in females, as compared to the entire female sample. Furthermore, no effect was evident in the subgroup of non-white females. Both of the above observations have significant social implications. Most beauty standards have been historically developed based on white facial features [43], and although beauty standards have evolved with western societies becoming more multi-racial, our finding entertains the thought that young white females might experience more pressure in meeting certain social standards of facial appearance. On the other hand, maybe the effect of facial shape on self-perceived facial attractiveness is mediated by the limited effect of other factors, such as skin texture. Coetzee et al. [44] studied a group of white and black individuals within a western society and observed that whites based their assessments of attractiveness primarily on facial shape, in contrast to blacks who were more influenced by skin tone. They connected their finding to the large variety of darker skin tones, to which whites are visually oblivious compared to blacks. Our results support this conclusion, since self-perceptions of non-white females were not affected by their facial shape. Another reason for this could be that the increased facial shape variation in the non-white females group compared to the white females’ group might have added noise to the outcomes failing to detect a significant effect. However, the absence of statistical significance on this test was definitive, based on the measured p-value, thus, not supporting this notion.

As mentioned before, the same observation was made here in white males; which may be subject to multifold interpretations. Males tend to have a higher self-esteem than females [45] and are more satisfied with their overall appearance [46]. This difference is unlikely to have a genetic or biological origin and seems to dissipate with age, since it is not seen in mature adults [45]. This universally seen phenomenon is rather a result of environmental factors that influence the development of self-esteem over a lifetime [47]. Western societies likely enable males to develop higher self-esteem than females, which in result affects more acquired social features. If so, it can be speculated that the effect of self-esteem on self-perceived attractiveness in young males overshadows any other, more objective feature. This is also supported by the comparison of VAS scores between males and females in our population, which showed that males gave significantly higher attractiveness ratings to themselves (P<0.001). The above considerations together with the reduced sample size of the white male sample might have made the detection of a significant effect in the specific sample impossible.

### Methodological considerations

The results of this study must be interpreted within the realm of the studied population and cannot be extrapolated to the general population. We have investigated a large group of young adults that were all highly educated, were born and had lived most of their lives in the United States. Despite their ethnic diversity, it may thus be assumed that their standards for facial attractiveness did not vary significantly. It must be noted that if the same study was repeated in an older population, the results might have been different due to changes in perception of attractiveness with age [48]. Here we did not report on the effect of age on the results, as an initial exploratory analysis revealed that it was not statistically significant.

In addition, participants were not able to look at their pictures prior to evaluating their facial attractiveness, which might have triggered a different response, had it been allowed. However, it was preferred to obtain more “genuine” answers that were not affected by the instant stimulus produced from prior exposure to their facial image.

Furthermore, the reliability testing did not include the image acquisition error related to the camera system since this has been found to be minimal (approximately 0.2mm) [49, 50]. Therefore, it was not considered to have a significant impact on the results.

### Significance and implications

This study provides novel and important information regarding the effect of facial morphology on self-perceived facial attractiveness. Self-assessments of body image and attractiveness are largely performed under the scope of psychosocial evaluations. Thus, the effect of objective features is often understated. Here we show that objective facial appearance is important when humans make decisions about their own facial attractiveness. In addition, we provide support to the notion that even in multicultural, modern societies, beauty stereotypes have changed little and continue to have a strong impact. Our findings are particularly insightful for plastic surgeons, maxillofacial surgeons, orthodontists and other specialists who are involved in treatments affecting patients’ facial appearance and particularly facial shape. Facial shape was identified as a factor related to facial appearance, and thus, as an important element to consider when aiming to improve facial appearance. The latter is shown to be a reason for patients to seek treatment and a factor that affects patient satisfaction from a given intervention. In addition, the results of this study provide helpful information to clinical psychologists interested in aspects of human perception, and are of interest for the general public as facial appearance is an important feature of everyday human interactions.

## Supporting information

S1 Fig Assessment of self-perceived attractiveness using a VAS scale. (TIF)

S2 Fig Assumptions of normality, linearity and homoscedasticity were tested with histograms, P-P plots and scatterplots. (TIF)

S3 Fig Scree plot displaying the % of variance explained by each PC. The first 16 PCs explained 80.4% of total shape variation in the sample and were selected in all subsequent analyses.
(TIF)

S4 Fig Bland-Altman Plot displaying the agreement in participant evaluations of self-perceived facial attractiveness (VAS scores), provided at two different time points. (TIF)

S5 Fig 3D PCA graph displaying facial variation among white individuals (in SD units), as explained by PC1 (20.8%), PC2 (12.6%) and PC3 (8.5%). The corresponding 3D facial morphings represent the average female (yellow) and male (blue) facial shapes. A best-fit superimposition (upper right) reveals the surface differences in facial shape between males and females and the color-map (lower right) displays the magnitude of those differences as distances of the female from the male face (positive: forward).
(TIF)

S6 Fig a. PCA graph displaying facial shape variation in white females (in SD units), as explained by PC1 (22%) and PC2 (10.1%). The corresponding 3D facial morphings show the shape extremes from -3 to +3 standard deviations of PC scores within each axis. b. Best fit superimpositions of shape transformations created from the Procrustes coordinates corresponding to -3SD and +3SD of PC scores within each axis from the average shape configuration in the white female population. The superimpositions display the direction of shape variation explained by each PC.
(TIF)

S7 Fig a. PCA graph displaying facial shape variation in white females (in SD units), as explained by PC3 (8.5%) and PC4 (6.7%). The corresponding 3D facial morphings show the shape extremes from -3 to +3 standard deviations of PC scores within each axis. b. Best fit superimpositions of shape transformations created from the Procrustes coordinates corresponding to -3SD and +3SD of PC scores within each axis from the average shape configuration in the white female population. The superimpositions display the direction of shape variation explained by each PC.
(TIF)

S8 Fig a. PCA graph displaying facial shape variation in white males (in SD units), as explained by PC1 (22%) and PC2 (11.3%). The corresponding 3D facial morphings show the shape extremes from -3 to +3 standard deviations of PC scores within each axis. b. Best fit superimpositions of shape transformations created from the Procrustes coordinates corresponding to -3SD and +3SD of PC scores within each axis from the average shape configuration in the white male population. The superimpositions display the direction of shape variation explained by each PC.
(TIF)

S9 Fig a. PCA graph displaying facial shape variation in white males (in SD units), as explained by PC3 (8.7%) and PC4 (6.7%). The corresponding 3D facial morphings show the shape extremes from -3 to +3 standard deviations of PC scores within each axis. b. Best fit superimpositions of shape transformations created from the Procrustes coordinates corresponding to -3SD and +3SD of PC scores within each axis from the average shape configuration in the white male population. The superimpositions display the direction of shape variation explained by each PC.
(TIF)

S10 Fig 3D PCA graph displaying facial variation among females (in SD units), as explained by PC1 (21%), PC2 (15.1%) and PC3 (9.1%). The corresponding 3D facial morphings represent the average white female (yellow) and non-white female (dark green) facial shapes. A best-fit superimposition (upper right) reveals the surface differences in facial shape between white and non-white females and the color-map (lower right) displays the magnitude of those differences as distances of the white female from the nor-white female face (positive: forward).
(TIF)

S11 Fig a. PCA graph displaying facial shape variation in non-white females (in SD units), as explained by PC1 (26.6%) and PC2 (15.9%). The corresponding 3D facial morphings show the shape extremes from -3 to +3 standard deviations of PC scores within each axis. b. Best fit superimpositions of shape transformations created from the Procrustes coordinates corresponding to -3SD and +3SD of PC scores within each axis from the average shape configuration in the non-white female population. The superimpositions display the direction of shape variation explained by each PC.
(TIF)

S12 Fig a. PCA graph displaying facial shape variation in non-white females (in SD units), as explained by PC3 (8.8%) and PC4 (6.3%). The corresponding 3D facial morphings show the shape extremes from -3 to +3 standard deviations of PC scores within each axis. b. Best fit superimpositions of shape transformations created from the Procrustes coordinates corresponding to -3SD and +3SD of PC scores within each axis from the average shape configuration in the non–white female population. The superimpositions display the direction of shape variation explained by each PC.
(TIF)

S13 Fig PCA graphs displaying facial shape variation (in SD units) in females (left) and males (right), regressed on self-perception of facial attractiveness (VAS scores). The color scale represents the progression of VAS scores from the least to the most attractive version of the female and male face, respectively.
(TIF)

S14 Fig PCA graphs displaying facial shape variation (in SD units) in white females, regressed on self-perception of facial attractiveness (VAS scores). The color scale represents the progression of VAS scores from the least to the most attractive version of the white female face.
(TIF)
