# Supplementary figures and images for: Facial shape affects self-perceived facial attractiveness
Source: PLoS One. 2021 Feb 3;16(2):e0245557. doi: 10.1371/journal.pone.0245557 (PMC7857636; doi:10.1371/journal.pone.0245557)

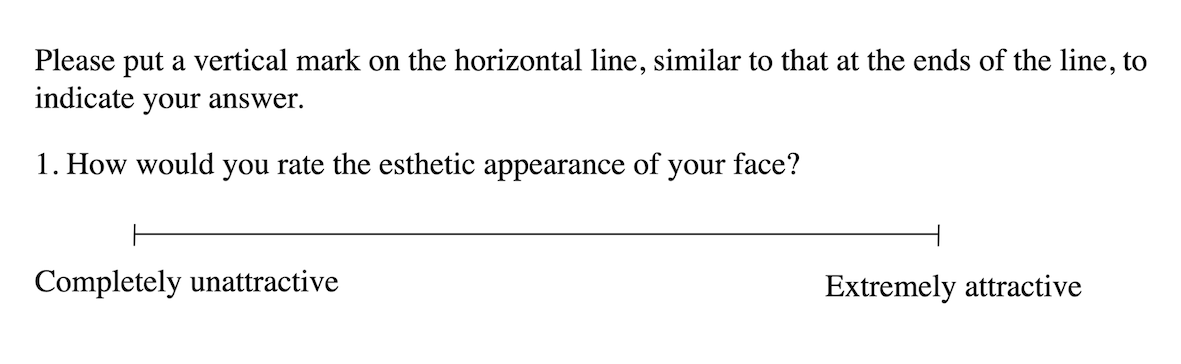

Supplement: S1 Fig — (TIF) [file pone.0245557.s001.tif]

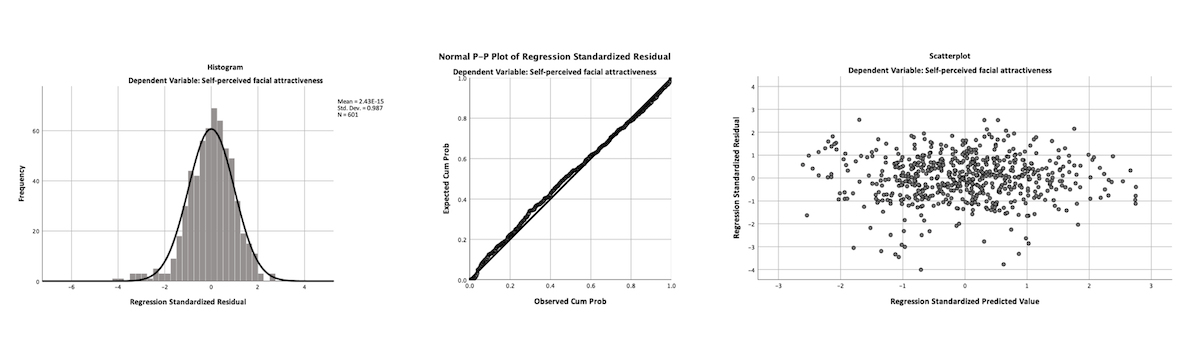

Supplement: S2 Fig — (TIF) [file pone.0245557.s002.tif]

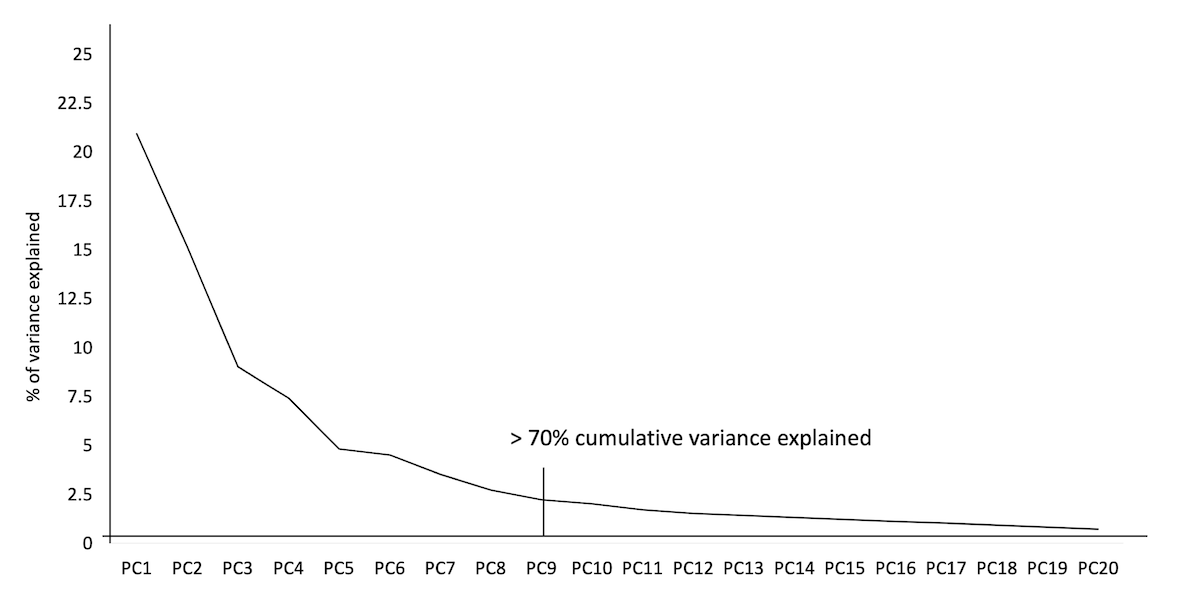

Supplement: S3 Fig — The first 16 PCs explained 80.4% of total shape variation in the sample and were selected in all subsequent analyses. (TIF) [file pone.0245557.s003.tif]

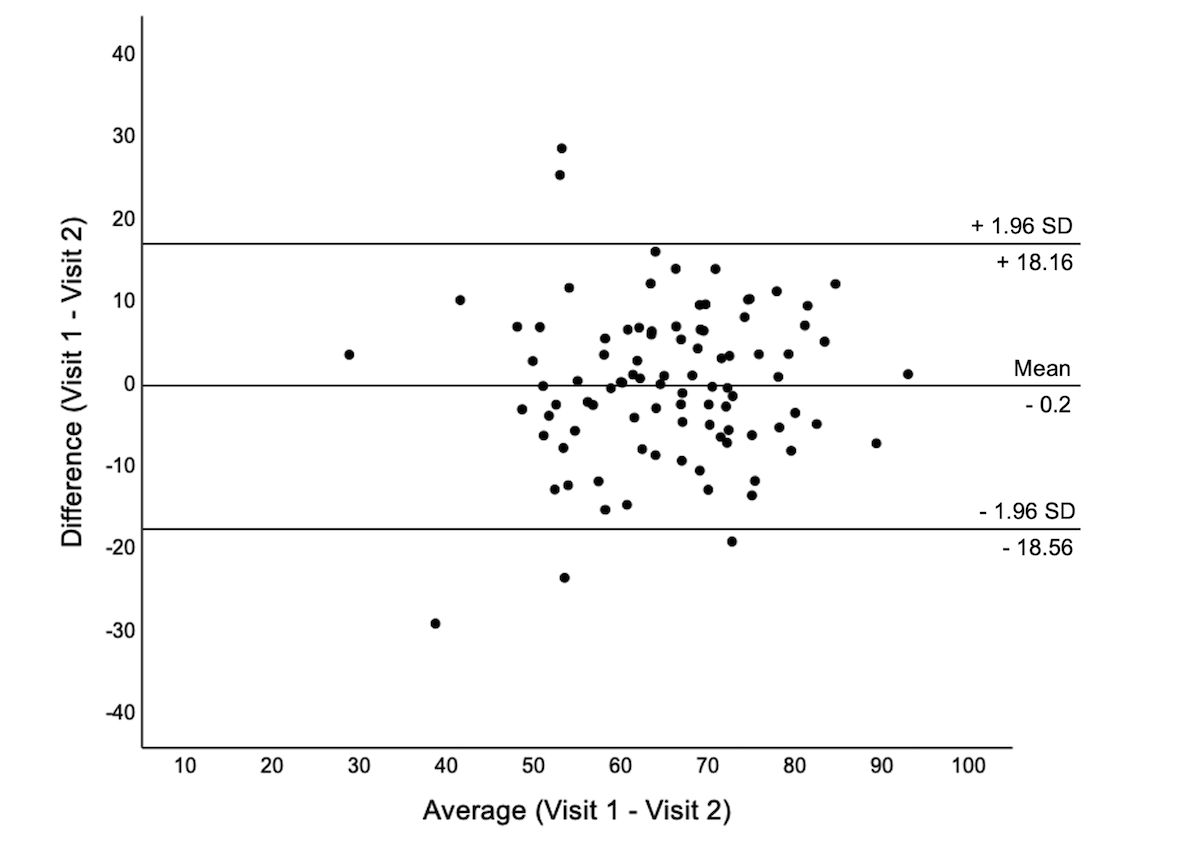

Supplement: S4 Fig — (TIF) [file pone.0245557.s004.tif]

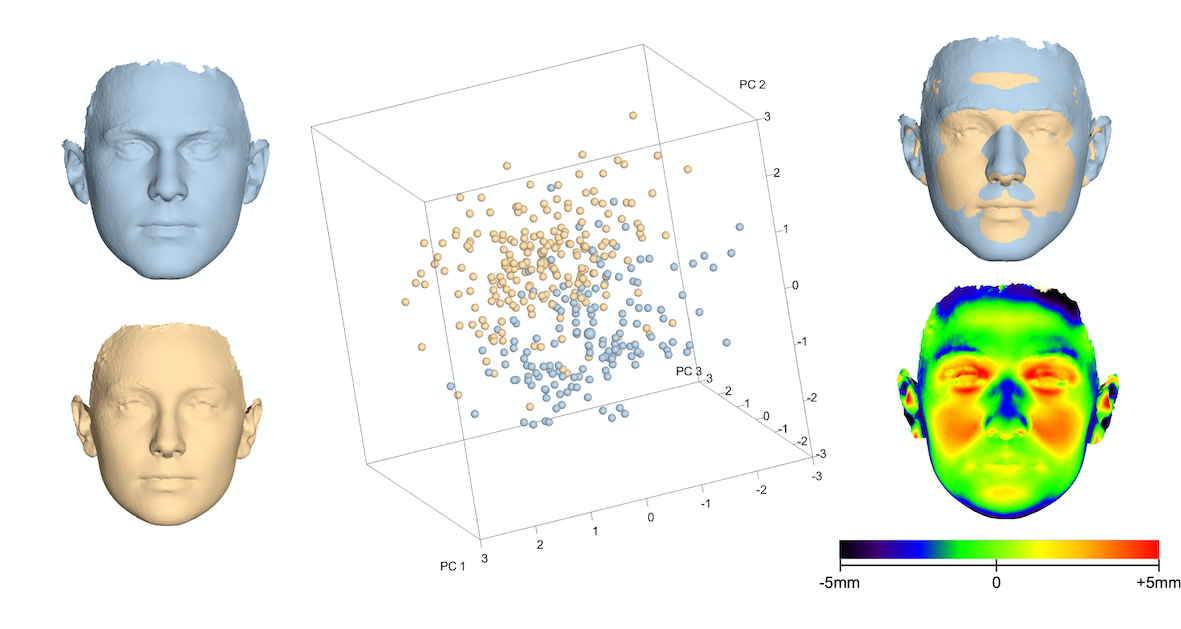

Supplement: S5 Fig — The corresponding 3D facial morphings represent the average female (yellow) and male (blue) facial shapes. A best-fit superimposition (upper right) reveals the surface differences in facial shape between males and females and the color-map (lower right) displays the magnitude of those differences as distances of the female from the male face (positive: forward). (TIF) [file pone.0245557.s005.tif]

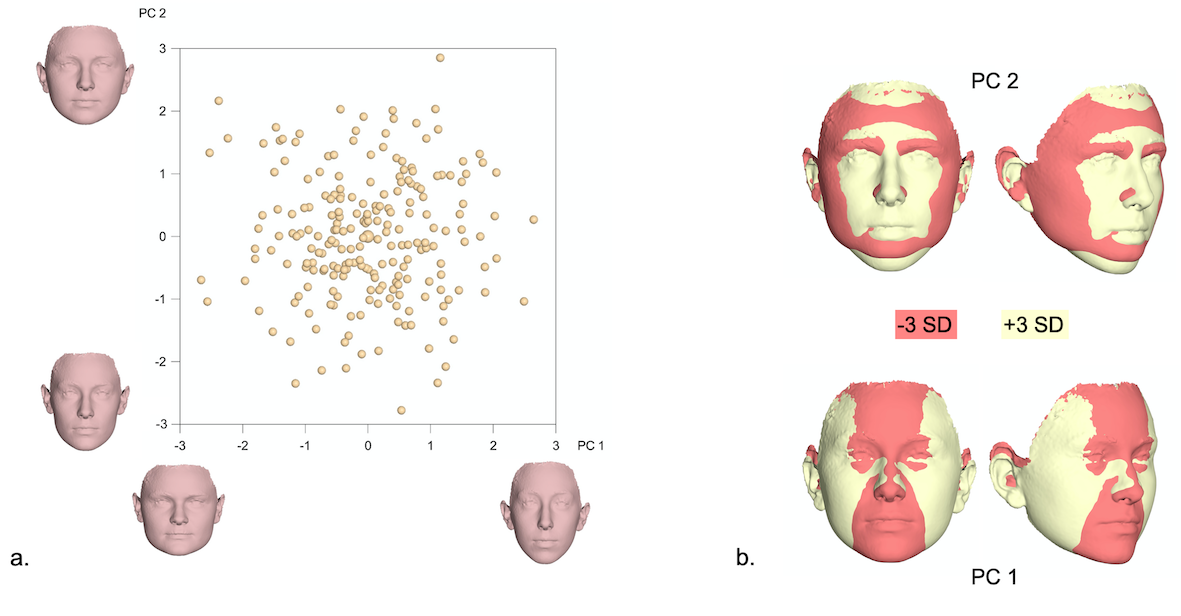

Supplement: S6 Fig — a. PCA graph displaying facial shape variation in white females (in SD units), as explained by PC1 (22%) and PC2 (10.1%). The corresponding 3D facial morphings show the shape extremes from -3 to +3 standard deviations of PC scores within each axis. b. Best fit superimpositions of shape transformations created from the Procrustes coordinates corresponding to -3SD and +3SD of PC scores within each axis from the average shape configuration in the white female population. The superimpositions display the direction of shape variation explained by each PC. (TIF) [file pone.0245557.s006.tif]

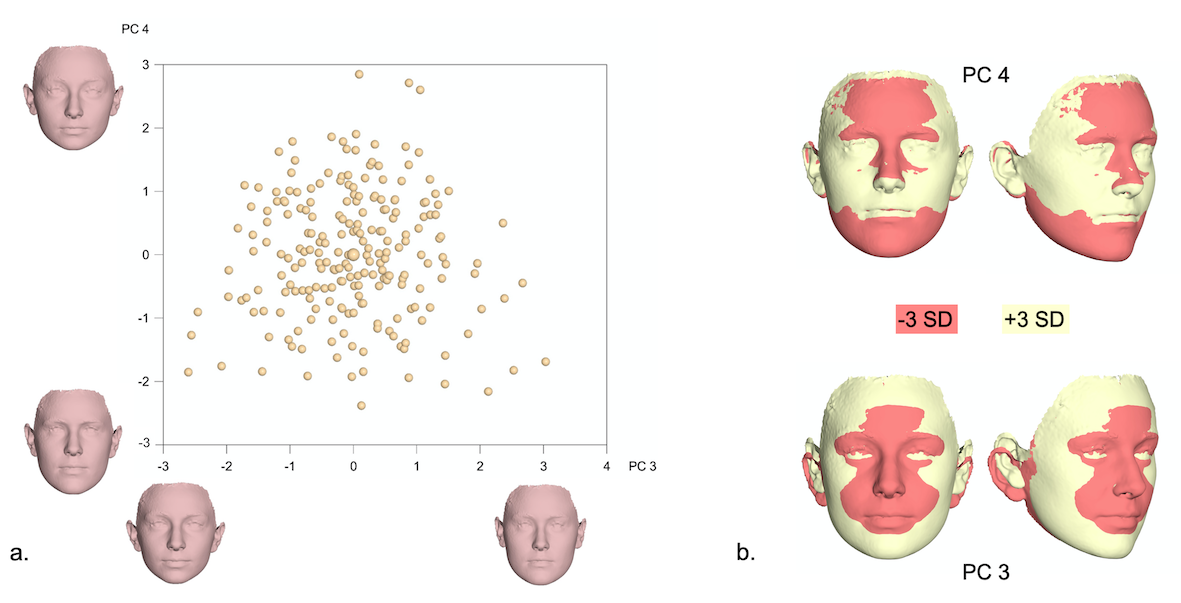

Supplement: S7 Fig — a. PCA graph displaying facial shape variation in white females (in SD units), as explained by PC3 (8.5%) and PC4 (6.7%). The corresponding 3D facial morphings show the shape extremes from -3 to +3 standard deviations of PC scores within each axis. b. Best fit superimpositions of shape transformations created from the Procrustes coordinates corresponding to -3SD and +3SD of PC scores within each axis from the average shape configuration in the white female population. The superimpositions display the direction of shape variation explained by each PC. (TIF) [file pone.0245557.s007.tif]

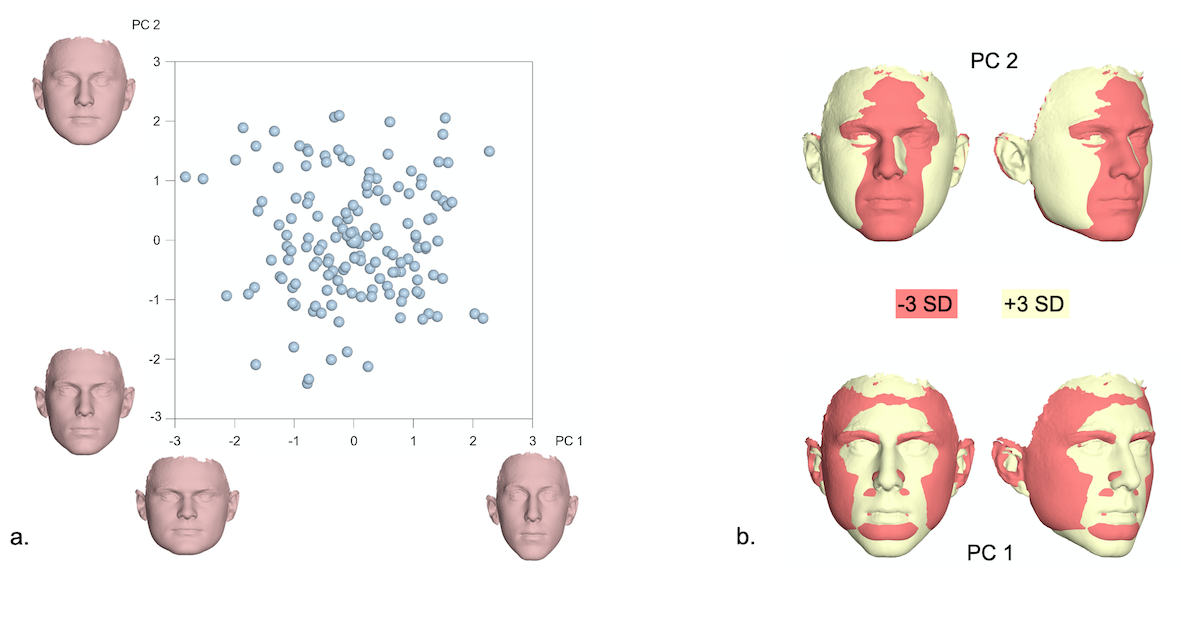

Supplement: S8 Fig — a. PCA graph displaying facial shape variation in white males (in SD units), as explained by PC1 (22%) and PC2 (11.3%). The corresponding 3D facial morphings show the shape extremes from -3 to +3 standard deviations of PC scores within each axis. b. Best fit superimpositions of shape transformations created from the Procrustes coordinates corresponding to -3SD and +3SD of PC scores within each axis from the average shape configuration in the white male population. The superimpositions display the direction of shape variation explained by each PC. (TIF) [file pone.0245557.s008.tif]

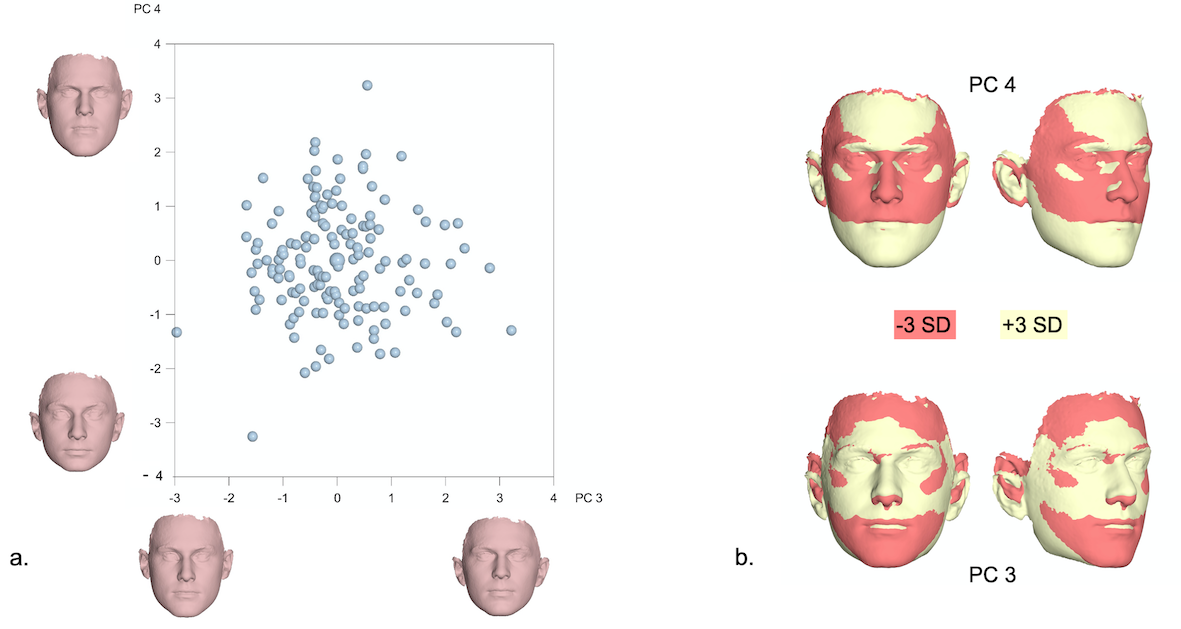

Supplement: S9 Fig — a. PCA graph displaying facial shape variation in white males (in SD units), as explained by PC3 (8.7%) and PC4 (6.7%). The corresponding 3D facial morphings show the shape extremes from -3 to +3 standard deviations of PC scores within each axis. b. Best fit superimpositions of shape transformations created from the Procrustes coordinates corresponding to -3SD and +3SD of PC scores within each axis from the average shape configuration in the white male population. The superimpositions display the direction of shape variation explained by each PC. (TIF) [file pone.0245557.s009.tif]

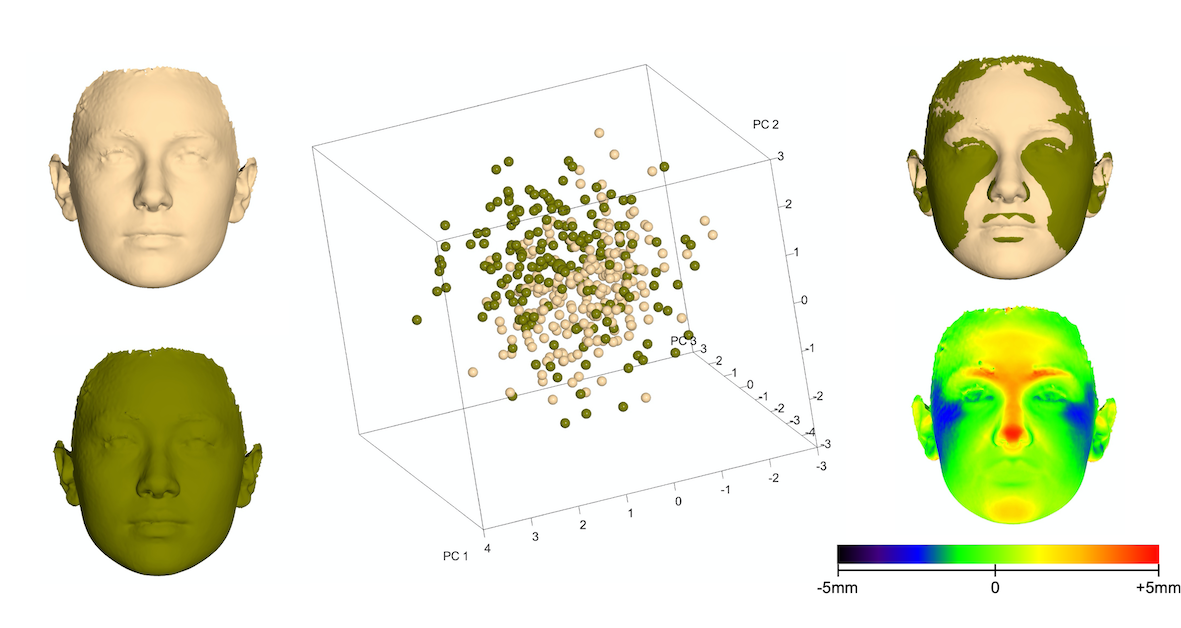

Supplement: S10 Fig — The corresponding 3D facial morphings represent the average white female (yellow) and non-white female (dark green) facial shapes. A best-fit superimposition (upper right) reveals the surface differences in facial shape between white and non-white females and the color-map (lower right) displays the magnitude of those differences as distances of the white female from the nor-white female face (positive: forward). (TIF) [file pone.0245557.s010.tif]

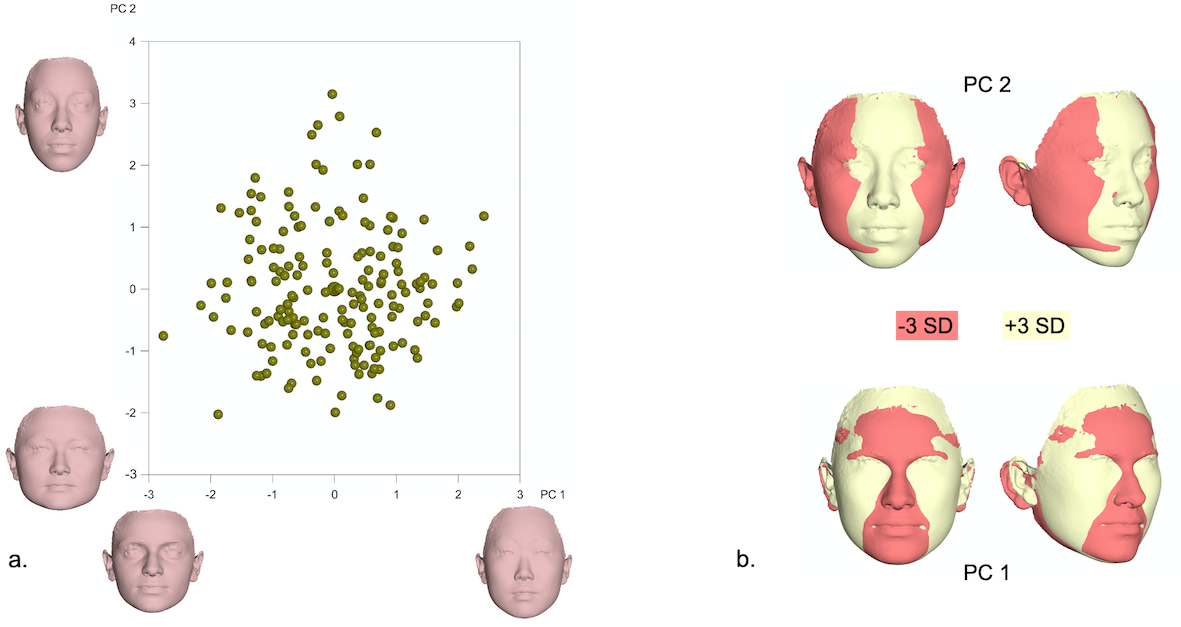

Supplement: S11 Fig — a. PCA graph displaying facial shape variation in non-white females (in SD units), as explained by PC1 (26.6%) and PC2 (15.9%). The corresponding 3D facial morphings show the shape extremes from -3 to +3 standard deviations of PC scores within each axis. b. Best fit superimpositions of shape transformations created from the Procrustes coordinates corresponding to -3SD and +3SD of PC scores within each axis from the average shape configuration in the non-white female population. The superimpositions display the direction of shape variation explained by each PC. (TIF) [file pone.0245557.s011.tif]

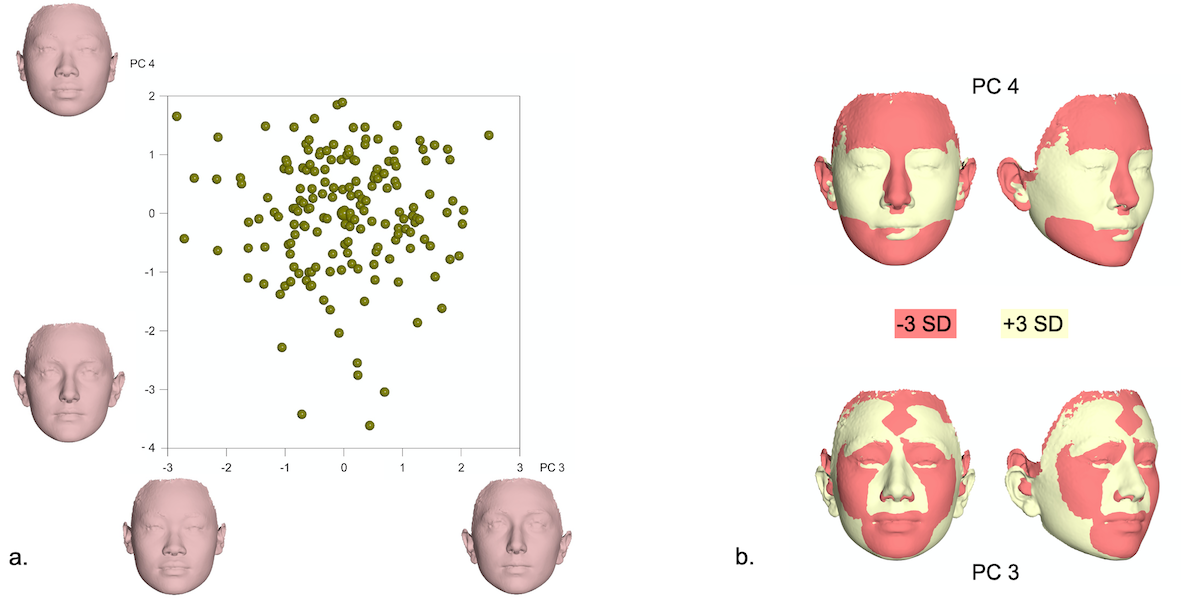

Supplement: S12 Fig — a. PCA graph displaying facial shape variation in non-white females (in SD units), as explained by PC3 (8.8%) and PC4 (6.3%). The corresponding 3D facial morphings show the shape extremes from -3 to +3 standard deviations of PC scores within each axis. b. Best fit superimpositions of shape transformations created from the Procrustes coordinates corresponding to -3SD and +3SD of PC scores within each axis from the average shape configuration in the non–white female population. The superimpositions display the direction of shape variation explained by each PC. (TIF) [file pone.0245557.s012.tif]

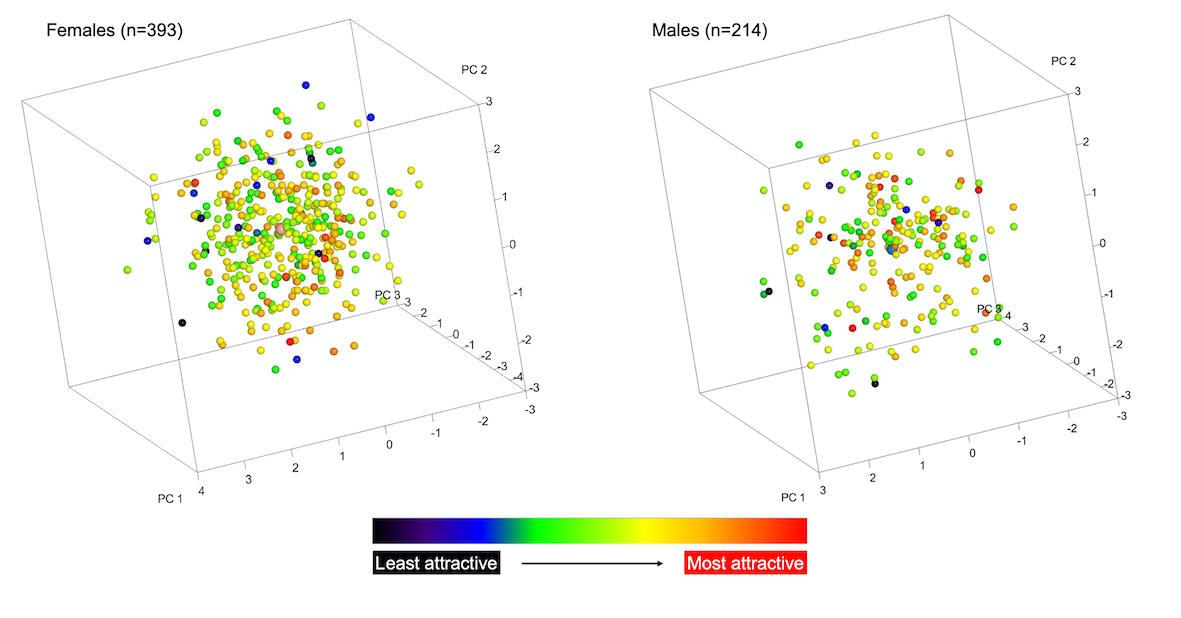

Supplement: S13 Fig — The color scale represents the progression of VAS scores from the least to the most attractive version of the female and male face, respectively. (TIF) [file pone.0245557.s013.tif]

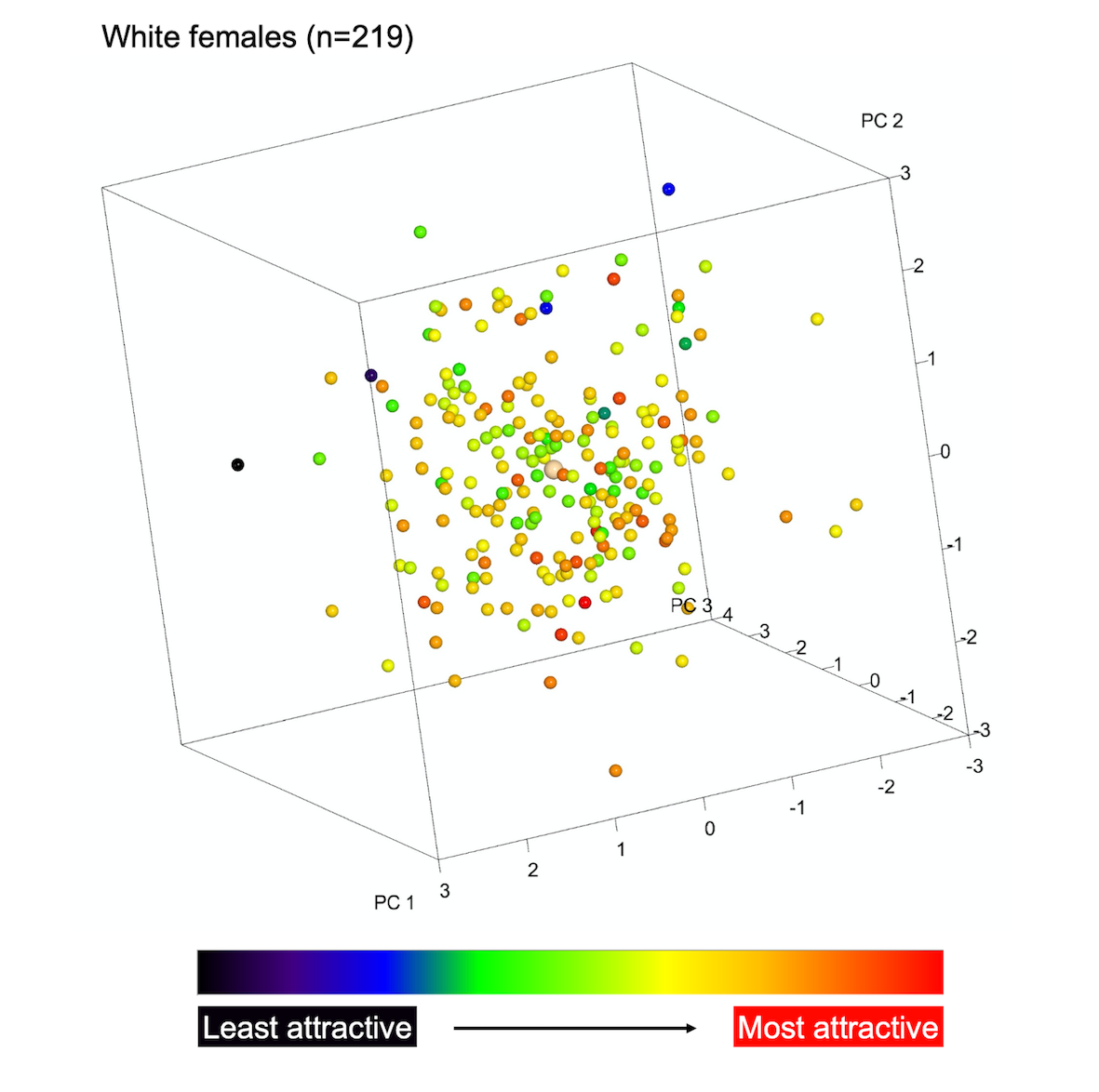

Supplement: S14 Fig — The color scale represents the progression of VAS scores from the least to the most attractive version of the white female face. (TIF) [file pone.0245557.s014.tif]
